# Photo-responsive degradable hollow mesoporous organosilica nanoplatforms for drug delivery

**DOI:** 10.1186/s12951-020-00642-1

**Published:** 2020-06-15

**Authors:** Jie Fan, Zhipeng Zhang, Yaru Wang, Shiting Lin, Shun Yang

**Affiliations:** School of Chemistry and Chemical Engineering, Jiangsu Normal University, Xuzhou, 221116 Jiangsu China

**Keywords:** Organosilica, Photo-responsive degradation, Drug delivery, Graphene oxide quantum dots, Hollow mesoporous materials

## Abstract

**Background:**

Stimulus-responsive degradable mesoporous organosilica nanoparticles (MONs) have shown great promise as drug carriers via enhancing the efficiency of drug delivery and accelerating the degradation of nanocarriers. However, it remains a great challenge to develop novel light-enabled spatial and temporal degradable MONs with both superior responsiveness for efficient anti-cancer drug delivery and safe exocytosis.

**Results:**

We report a novel photo-responsive degradable hollow mesoporous organosilica nanoplatform (HMONs@GOQD). The platform is based on organosilica nanoparticles (HMONs) containing singlet oxygen (^1^O_2_)-responsive bridged organoalkoxysilanes and wrapped graphene oxide quantum dots (GOQDs). The unique hollow mesoporous structure of the HMONs guarantees an excellent drug loading and release profile. During light irradiation, ^1^O_2_ produced by the GOQDs leads to the degradation of the organosilica nanoparticles, resulting in enhanced local drug release.

**Conclusions:**

We carried out in vitro and in vivo experiments using DOX as a model drug; DOX-HMONs@GOQDs exhibited high biocompatibility, accelerated degradation, and superior therapeutic efficacy during light irradiation, indicating a promising platform for clinical cancer therapy.

## Background

With the development of nanotechnology, smart drug delivery systems (DDS) have been widely employed to overcome the side effects of anti-cancer drugs in chemotherapy [1–4]. Numerous small-scale DDS (nanoparticles and microparticles) are fabricated using various biocompatible materials, such as organic molecules, lipids, polymers, and inorganic nanomaterials. They enables the targeted delivery of drugs to tumors owing to the engineered surfaces of the DDS and the effect of their enhanced permeability and retention (EPR) of tumor cells [5–10]. However, as well as unique chemical stability, a high surface area, tunable size, and porosity, ideal DDS have biorelated degradability and clearance as essential features. Some DDS comprising particles with sizes larger than 10 nm can be taken up by the liver and spleen, which gives rise to long-term toxicity concerns [11–15].

Although some DDS derived from polymeric nanoparticles (NPs) with special organic chemical structures, such as poly(lactic acid) [16–19], poly(lactide-*co*-glycolide) [20–24], and poly(ε-caprolactone) [25–28], are degradable in biological environments, inefficient drug loading and mechanical and chemical instability still impede their clinical translation. Therefore, nanoparticles with the advantages of both organic (biodegradability) and inorganic (unique chemical stability, high surface area, tunable size, and porosity) nanomaterials would pave the way to the preparation of ideal drug delivery systems [29–36].

Mesoporous organosilica nanoparticles (MONs)based on bridged organoalkoxysilanes with two or more alkoxysilyls have been designed due to the advantages of the siloxane matrix, such as a defined porous framework and specific organic functionalities [37–42]. The unique high surface area and mesoporous structure of MONs contribute to their drug loading efficiency. To accelerate the degradation of MONs, and address the long-term toxicity concerns, stimuli-responsive bridged organoalkoxysilanes have also been introduced to prepare biodegradable MONs. For instance, disulfide bridges have been incorporated into the walls of MONs to create redox-responsive biodegradable nanoparticles, which rely on the cleavage of sulfide bridges by intracellular bioreducing agents such as glutathione tripeptides [43–48]. Oxamide–phenylene bridges, which are biodegradable in the presence of trypsin proteins, have also been reported [49]. Such biodegradable MONs-based DDS are safe with regard to exocytosis, overcome the long-term toxicity concerns, and release the loaded drug more readily.

Compared with the stimulus provided by the specific chemical properties of the cellular environment, an external light source enables better spatiotemporal dosing of the loaded drug, and more responsive on-demand degradation of the DDS. Some effective DDS using MONs as drug containers have been developed for photo-responsive drug delivery, however, the degradation of the MONs themselves relies on the cellular environment, which is a relatively slow process [50, 51]. Therefore, the design and fabrication of novel photo-induced biodegradable MONs would not only speed up the release of the loaded drugs, but would also accelerate the safe exocytosis of the DDS. This would be significant for the future clinical transformation of MONs-based DDS.

**Scheme 1:**
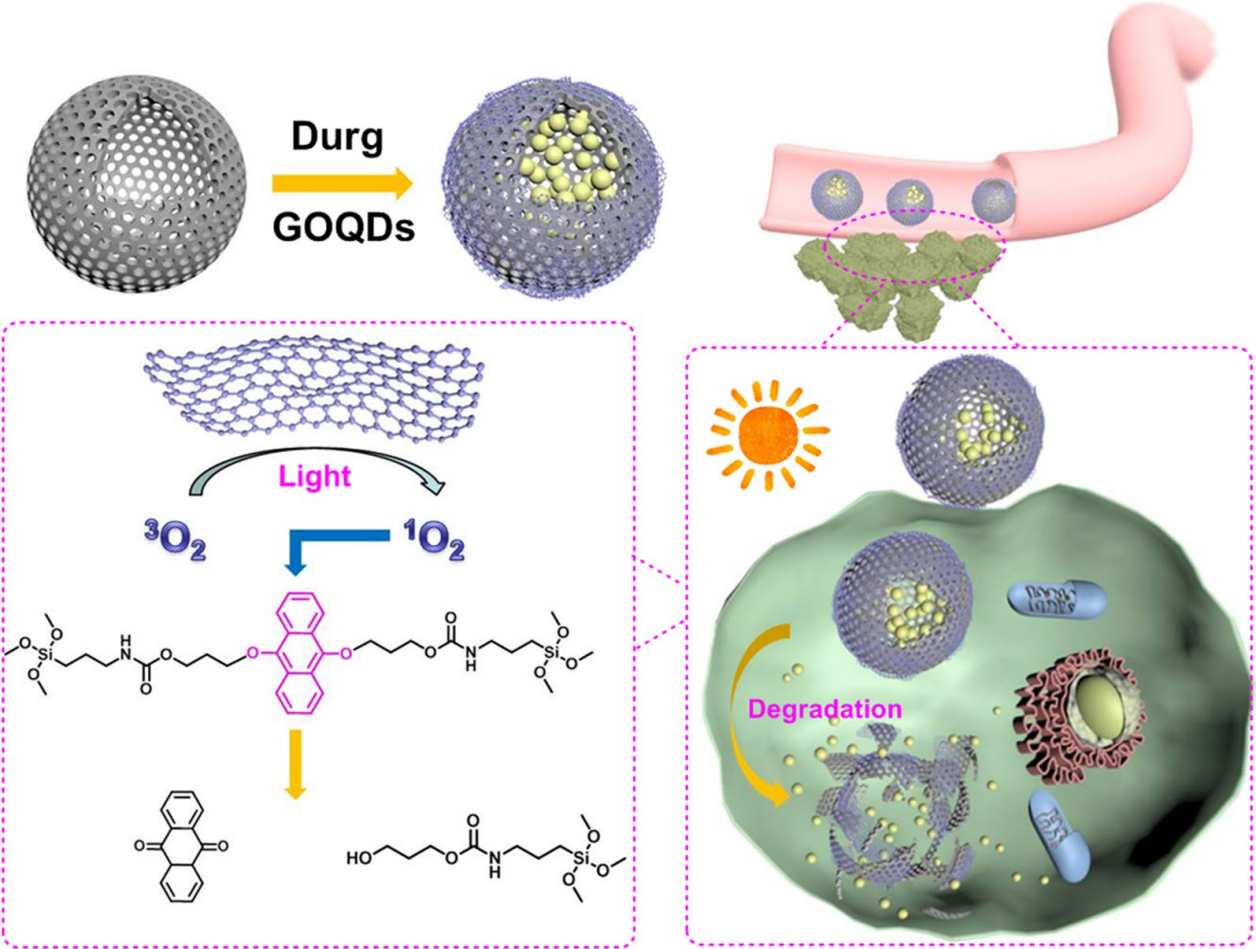
Schematic illustration of organosilica nanoplatform (HMONs@GOQDs) for cancer threapy

In the present study, we designed and fabricated a novel photo-responsive hollow degradable mesoporous organosilica nanoplatform for anti-cancer drug delivery. The nanoplatform was based on singlet oxygen (^1^O_2_)-responsive bridged organoalkoxysilanes (from a 9,10-dialkoxy-anthracene (DN)-based precursor), and wrapped graphene quantum dots (GOQDs). The hollow structures of the organosilica nanoparticles (HMONs) used for the platform were intended to provide high drug loading content, and the modified GOQDs on the surfaces of the MONs were included to delay drug release in normal tissue. During light irradiation, the GOQDs produce ^1^O_2_ in a significantly high quantum yield [52–54], leading to the cleavage of ^1^O_2_-responsive bridges and the degradation of the nanoplatform (Scheme 1). The loaded anti-cancer drug is then released efficiently to destroy the tumor cells. Furthermore, degradability and clearance of this photo-responsive hollow degradable mesoporous organosilica nanoplatforms (HMONs@GOQDs) are appealing for clinical trials to overcome long-term toxicity.

## Materials and methods

### Materials

*N*-hydroxysulfosuccinimide (NHS, 98%), *N*-(3-dimethylaminopropyl)-*N*′-ethylcarbodiimide hydrochloride (EDC, 99%), hexa-decyltrimethylammonium bromide (CTAB, > 99.0%), Tetraethoxysilane (TEOS), 3-Chloro-1-propanol (98%), Anthraquinone (98%), Adogen 464, 3-Isocyanatopropyltriethoxysilane (95%) and (3-aminopropyl)triethoxysilane (APTES) were purchased from Energy Chemical Co., Ltd (Shanghai, China). Ammonia (25.0–28.0%), nitric acid (65.0–68.0%) and sulfuric acid (95.0–98.0%) were purchased from Aladdin Reagent (Shanghai, China). MTT and RPMI-1640 were purchased from Sigma-Aldrich (St Louis, MO, USA). Other reagents were commercially available and used as received.

Breast cancer line 4T1 cells were originally obtained from the Type Culture Collection of the Chinese Academy of Sciences (Shanghai, China) and cultured as monolayers in a RPMI-1640 medium supplemented with 10% heat-inactivated fetal bovine serum at 37 °C in a humidified incubator (5% CO_2_ in air, v/v).

Male nude mice (4 weeks old, ≈ 16 g) were fed in the specific pathogen free (SPF) animal room at 20 °C for 1 week prior to use.

### Synthesis of graphene oxide quantum dots (GOQDs) and characterization

Graphene oxide quantum dots were synthesized by a modified strong-acid oxidation method. Typically, graphite powder (900 mg) were dispersed in concentrated sulfuric acid (100 mL, 95.0–98.0%), which was stirred for 1 h followed by sonication for 30 min. The resultant suspension was poured into a three-neck round-bottom flask which contained mixed acids (80 mL sulfuric acid (95.0–98.0%) and 60 mL nitric acid (65.0–68.0%). After 24 h of refluxing at 100 °C, the mixture was cooled to room temperature and a required amount of distilled water was slowly added. The pH was adjusted to ~ 8 with sodium bicarbonate. During this period, the reaction suspension was filtered for 3 times to remove the precipitated salt. Afterwards, the residual solution was collected by decantation with filter paper. The filtrate was dialysed in a dialysis bag (MWCO 3500 Da) against distilled water for more than 7 days to remove excess salt. The suspension was freeze-dried to obtain GOQDs powder with a yield of ca. 5%.

### Synthesis of 9,10-dialkoxyanthracene (DN), possessing triethoxysilyl groups (DN precursor)

Briefly, 10 g 3-Chloro-1-propanol and 60 g sodium iodide were added into 100 mL acetone, the mixture was heated to 60 °C under nitrogen for 24 h, after filtration and evaporation, a 1:1 mixture of diethyl ether-hexane (100 mL) was added and stirred for 10 min at 25 °C and then filtered. The solution was washed with a dilute sodium thiosulfate solution followed by water and then brine. After the evaporation of organic solution, 3-Iodo-1-propanol was achieved. Yield 12.8 g, 67%; ^1^HNMR (400 MHz, CDCl_3_), δ (ppm): 3.73 (t, 2H, CH_2_OH), 3.28 (t, 2H, CH_2_I), 2.07 (m, 2H, CH_2_CH_2_CH_2_).

9,10-Anthraquinone (2.57 g), Na_2_S_2_O_4_ (4.3 g) and Adogen 464 (4.64 g) were added into nitrogen-saturated water (150 mL) and CH_2_Cl_2_ (150 mL). After stirring for 5 min at 25 °C, NaOH (4.94 g) was added, and then 3-iodo-1-propanol was added dropwise. The mixture was stirred for 12 h at 25 °C. Then the phases were separated and the organic phase was washed with water for 3 times. The solution volume was reduced to 40 mL in a rotary evaporator and the product was precipitated overnight at − 20 °C. The solid was purified by column chromatography with a mixture of ethyl acetate-CH_2_Cl_2_ (1/4, v/v). Yield 1.13 g, 28%; ^1^HNMR (400 MHz, DMSO-d_6_), δ (ppm): 8.24 (dd, J = 6.8, 3.2 Hz, 4H, anthracene), 7.52 (dd, J = 6.8, 3.2 Hz, 4H, anthracene), 4.63 (t, J = 5.2 Hz, 2H, CH(O)), 4.18 (t, J = 6.5 Hz, 4H, OCH_2_CH_2_), 3.76 (m, CH_2_CH_2_OH, 4H), 2.11 (m, CH_2_CH_2_CH_2_, 4H,).

9, 10-Bis(3-hydroxypropyloxy)anthracene (0.5 g), 3-Isocyanatopropyltriethoxysilane (0.38 g) and Triethylamine (0.155 g) were added into 100 mL ethanol. Then the mixture was refluxed at 80 °C for 12 h. and the product was purified by column chromatography with a mixture of ethyl acetate-CH_2_Cl_2_ (1/4, v/v). Yield 0.75 g, 85.2%.

### Synthesis of DN group based hollow mesoporous organosilica nanoparticles (HMONs)

DN group based hollow mesoporous organosilica nanoparticles (HMONs) was synthesized according to the reported procedure [55]. Briefly, CTAB aqueous solution (5 g, 10 wt %) and TEA aqueous solution (0.2 g, 10 wt %) were mixed (pH = 9–10), the solution was then stirred in an oil bath at 95 °C for 10 min before 1 mL of TEOS was added dropwise. After 1 h, the silica core was generated. 0.25 mL TEOS and 0.25 g DN precursor were then added and the mixture was heated to 60 °C and stirred for 4 h. And the product was collected by centrifugation and washed with ethanol three times. To remove the silica core, the solid was redispersed in 100 mL water, and 2 mL ammonia (25.0–28.0%) were added, and the etching process lasted for 3 h at 95 °C. And HMONs were finally collected by centrifugation and washed with water.

### Synthesis of photo-responsive biodegradable hollow mesoporous organosilica nanoplatforms (HMONs@GOQDs)

9 mg HMONs were dispersed in 5 mL ethanol, then 0.5 mL of (3-aminopropyl) triethoxysilane (APTES) was added. The obtained suspension was stirred for 24 h at 25 °C and collected by centrifugation, washed with ethanol several times and dried under vacuum to give HMONs-NH_2_. GOQDs aqueous solution (1 mg mL^−1^, 2 mL) was mixed with EDC (20 mg), NHS (40 mg) and 2 mL H_2_O, and the mixture was stirred for 1 h at 25 °C. Then the HMONs-NH_2_ aqueous solution (3 mg mL^−1^, 3 mL) were added to stir for another 12 h. HMONs@GOQDs nanohybrids were extracted with centrifugation and then washed with water for 3 times.

To prepare DOX-HMONs@GOQDs, GOQDs aqueous solution (1 mg mL^−1^, 2 mL) was mixed with EDC (20 mg), NHS (40 mg) and 2 mL H_2_O, and the mixture was stirred for 1 h at 25 °C. Then the HMONs-NH_2_ aqueous solution (3 mg mL^−1^, 3 mL) and DOX solution (5 mg mL^−1^ in DMSO, 200 µL) were added to stir for another 12 h. DOX-HMONs@GOQDs were extracted with centrifugation and then washed with water for 3 times. The DOX loading efficiency was calculated as follows:

$$ {\text{Loading efficiency }} = \, {{({{\text{C}}}_{0} {\text{V}}_{0} - {\text{C}}_{{\text{t}}} {{\text{V}}}_{{\text{t}}} )} \mathord{\left/ {\vphantom {{({\text{C}}_{0} {\text{V}}_{0} - {{\text{C}}}_{{\text{t}}} {{\text{V}}}_{{\text{t}}} )} {{{\text{C}}}_{0} {\text{V}}_{0} }}} \right. \kern-0pt} {{\text{C}}_{0} {\text{V}}_{0} }} $$

where C_0_ and V_0_ are the concentration and volume of the added DOX, respectively, and C_t_ and V_t_ are the concentration and volume of free DOX after the centrifugation and washing, respectively.

### Degradation evaluation of HMONs@GOQDs

To determine the structural evolution of HMONs@GOQDs under light irradiation, HMONs@GOQDs were dispersed in simulated body fluid (SBF) at 37 °C under slow stirring. Then the solution was irradiated with a Violet Blue Laser (365 nm, 0.8 W m^−2^) for 10 min at 30 min interval. The partially degraded HMONs@GOQDs was collected by centrifugation and directly observed by TEM characterization. Also the particle-size distribution of HMONs@GOQDs and supernatant was further analyzed by means of dynamic light scattering (DLS).

### Degradation assay of HMONs@GOQDs at cell level

To study the intracellular degradation behavior of HMONs@GOQDs, HMONs@GOQDs (100 μg mL^−1^ in PBS, pH 7.4) were co-incubated with 4T1 cells, and then irradiated by light for 10 min at 30 min interval. After different incubation durations, the cells were harvested, fixed, and sectioned for bio-TEM characterization.

### In vitro cytotoxicity

4T1 cells were seeded into 96-well cell culture plates at 1 × 10^4^ per well incubated overnight at 3 °C in a humidified incubator, and then incubated with HMONs@GOQDs or DOX-HMONs@GOQDs at certain concentrations for 4 h. After removal of nanoparticles, cells were transferred into fresh media and irradiated by light for certain times. The cells were then incubated at 37 °C for additional 24 h before the standard methyl thiazolyl tetrazolium (MTT, Sigma Aldrich) assay.

### In vivo experiments

To develop the tumor model, 4T1 cells (1 × 10^6^) suspended in 50 μL of PBS were subcutaneously injected into the leg of each male athymic nude mice (4 weeks old). Then mice were divided into three groups (n = 4 per group) for various treatments: (1) ca. 50 μL of Saline + light; (2) DOX-HMONs@GOQDs; (3) DOX-HMONs@GOQDs + light (once every 2 d at a 5 mg kg^−1^ DOX-equivalent dose), and the light (365 nm, 0.8 W m^−2^) were conducted for 12 h after the injection (30 min/12 h, 2 min intervals for each 1 min irradiation). The tumor size were measured by a caliper every other day and calculated as the volume = (tumor length) × (tumor width)^2^/2. And the relative tumor volumes were calculated as V/V_0_ (V_0_ was the initial tumor volume).

## Results and discussion

**Scheme 2:**
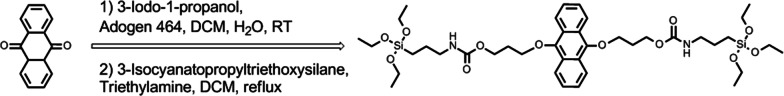
Synthesis of the 9,10-dialkoxy-anthracene (DN)-based precursor

We synthesized the DN-based precursor according to the route outlined in Scheme 2. The chemical structure of the precursor was confirmed by ^1^HNMR spectroscopy, as shown in Additional file 1: Fig. S1.

**Fig. 1:**
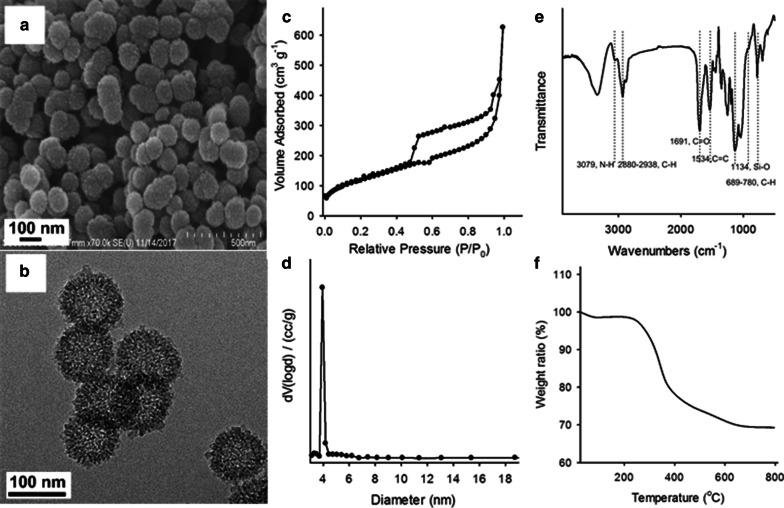
Characterization of the organosilica nanoparticles (HMONs). **a** Scanning electron microscopy (SEM) images of the HMONs; **b** transmission electron microscopy (TEM) images of the HMONs; **c** N_2_ adsorption and desorption pattern of the HMONs; **d** pore size distributions of the HMONs; **e** Fourier-transform infrared (FTIR) spectrum of the HMONs; and **f** thermogravimetric analysis (TGA) curves of the HMONs

We synthesized the HMONs via sol–gel methods using SiO_2_ as the hard template based on a “structure difference-based selective etching” strategy [55]. The MONs layer was readily coated on the surface of the silica template owing to its similarity to tetraethyl orthosilicate (TEOS) in the formation of the silica core. After the etching process, HMONs with high dispersity and well-defined hollow mesoporous structures were obtained. The scanning electron microscopy (SEM) and transmission electron microscopy (TEM) images shown in Fig. 1a, b and Additional file 1: Fig. S2 confirm the hollow structure of HOMs. The mesoporous structure was also confirmed by the N_2_ absorption–desorption isotherms. The Brunauer–Emmett–Teller (BET) surface area of the HMONs was 425.662 m^2^ g^−1^, and the pore size was distributed at ~ 3.94 nm (Fig. 1c, d, respectively). The high surface area is beneficial to the drug-loading capacity, and the mesopores enable drug loading and release.

To demonstrate the presence of DN groups in the HMONs shells, Fourier-transform infrared (FTIR) spectra was carried out. As shown in Fig. 1e. the peak located at 1534 cm^−1^ is attributable to the C=C of the anthracene group, and the incorporation of organic bridges is revealed by νN–H at 3079 cm^−1^ and νC=O at 1691 cm^−1^, as well as by aliphatic and aromatic C-H stretching modes. The high degree of condensation of the siloxanes is confirmed by the shift of νSi–O at 1134 cm^−1^. We also used TGA to demonstrate the high organic content of the nanocomposites. The two significant weight losses presented at 220 and 400 °C were attributable to the thermal decomposition of the aliphatic chains and anthracene groups in the organic bridges, respectively, resulting in a total weight loss of 31%.

GOQDs are novel quantum dots, which have received considerable attention because of their excellent optical properties, favorable chemical stability, and low toxicity. They also provide a good platform for the production of ^1^O_2_ with a sufficiently high quantum yield for photodynamic therapy. Furthermore, GOQDs are easily modified due to the abundant hydroxyl and carboxyl groups on their surfaces [56–59]. Therefore, the GOQDs were prepared. First, we oxidized graphite powder in concentrated H_2_SO_4_ and HNO_3_. The particle size of the powder decreased dramatically after alkali treatment, and we isolated the resulting ultrafine GOQDs by dialysis [60]. As shown in Additional file 1: Fig. S3a, the GOQDs were approximately 10 nm in size. The emission spectrum of the GOQDs excited by 375-nm light also demonstrated the successful synthesis (Additional file 1: Fig. S3b). Under light irradiation, the GOQDs could transfer the photon energy to ^3^O_2_ leading to the generation of ^1^O_2_, and to assess the ability of GOQDs to generate ^1^O_2_, 9,10-anthracenediyl-bis(methylene) dimalonic acid (ABDA) was used as the trapping agent [61]. As illustrated in Additional file 1: Fig. S7, the absorbance of the ABDA decreased gradually with prolonged irradiation time, indicating the degradation of ABDA by ^1^O_2_ generated by GOQDs. The absorption intensity of the ABDA decreased to approximately 70% after irradiation at 365 nm (0.8 W/cm^2^) [53] for 10 min. In addition, the Singlet Oxygen Sensor Green (SOSG) measurements shown in Additional file 1: Fig. S8 also confirms the good ^1^O_2_ generation efficacy of GOQDs.

To coat the surface of the HMONs with GOQDs, we first modified the HMONs with amino groups using aminopropyltriethoxysilane via the Si–OH groups on their surfaces. We then activated the GOQDs using EDC/NHS chemistry, and attached them to the surfaces of the HMONs by direct covalent bonds. The resultant HMONs@GOQDs were characterized by SEM, TEM, dynamic light scattering (DLS), and UV–vis absorption analysis.

**Fig. 2:**
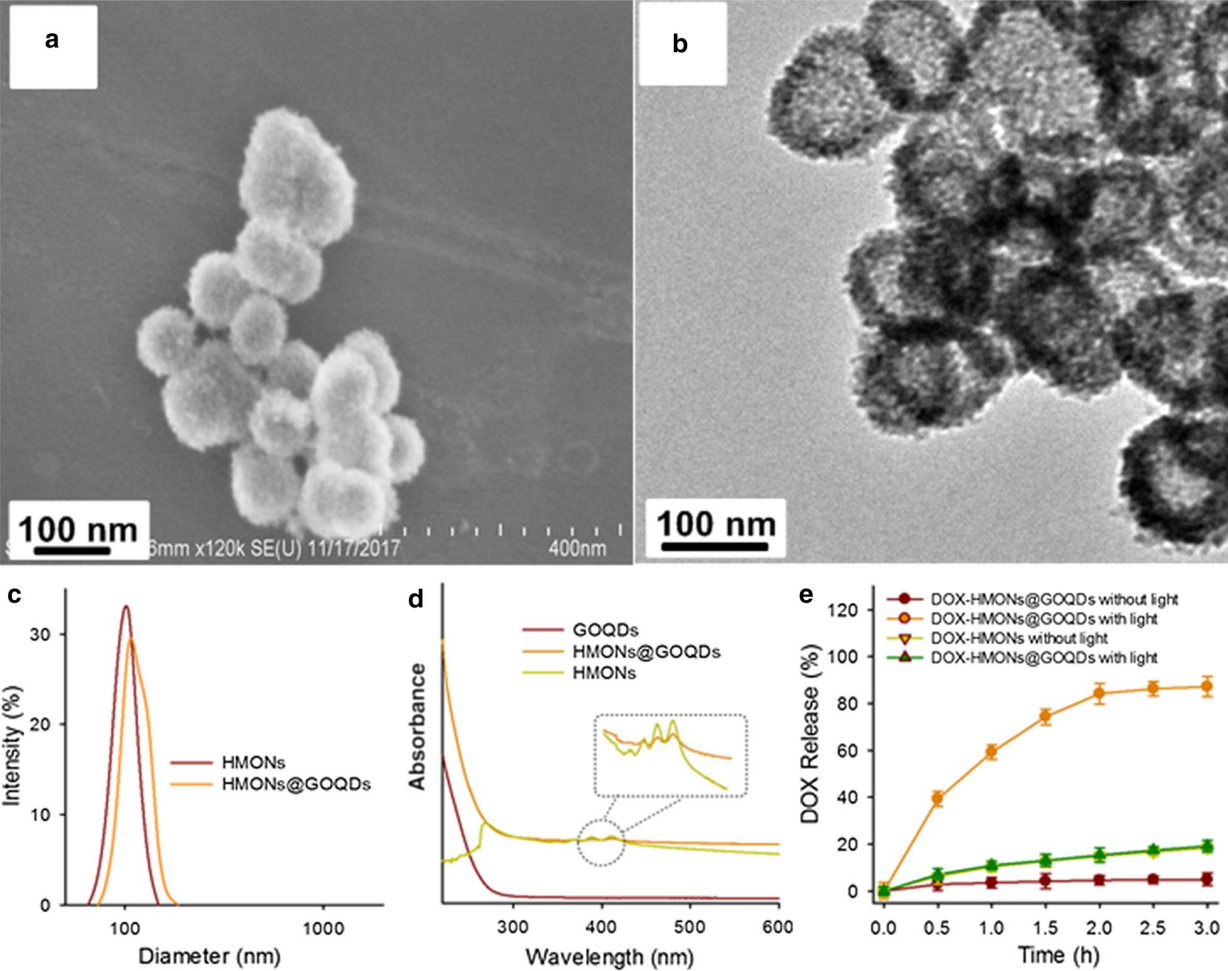
Characterization of HMONs@GOQDs. **a** Scanning electron microscopy (SEM) images of the HMONs@GOQDs; **b** transmission electron microscopy (TEM) images of the HMONs@GOQDs; **c** size distribution of the organosilica nanoparticles (HMONs) and HMONs@GOQDs; **d** UV–vis adsorption of the graphene oxide quantum dots (GOQDs), HMONs, and HMONs@GOQDs; **e** percentages of DOX released from DOX-HMONs and DOX-HMONs@GOQDs with or without light irradiation

The HMONs@GOQDs retained their spherical morphology after the coating process. Furthermore, they had good dispersibility in aqueous solution owing to their abundant –OH and –COOH groups, and retained their high dispersion stability in phosphate-buffered saline (PBS), even after 6 h (Additional file 1: Fig. S4). The high dispersibility of the HMONs@GOQD particles is apparent from the SEM image of a diluted sample (Additional file 1: Fig. S5), and could be further improved by surface modification with polyethylene glycol [62]. However, because the contrast arising from GOQDs is much lower than that from HMONs, it is difficult to directly observe the conjugation between the HMONs and GOQDs (Fig. 2a, b). The DLS of the HMONs and HMONs@GOQDs confirms the conjugation, as shown in Fig. 2c; the difference in particle size was entirely due to the coating of the GOQDs. The DLS of the HMONs@GOQDs also confirmed their favorable dispersity in aqueous solution. The UV–vis spectra of the GOQDs, HMONs, and HMONs@GOQDs are shown in Fig. 2d; the new absorption peak at 210–220 cm^−1^ attributable to HMONs@GOQDs proves successful conjugation owing to the presence of the characteristic GOQD peaks. We also determined the carbon contents of the HMONs and HMONs@GOQDs using a CHNS/O analyzer (PE 2400 II, Perkin Elmer, USA); the obtained carbon contents were 23.8 wt % and 32.9 wt %, respectively. This increased carbon content further confirmed the presence of GOQDs. The weight percentage of GOQDs to HMONs was calculated to be approximately 8.3 wt %. The BET surface area of the HMONs@GOQDs was 527.446 m^2^/g (Additional file 1: Fig. S6), which was higher than that of HMONs.

The production of ^1^O_2_ by HMONs@GOQDs was also investigated, and the reduced absorbance of ABDA confirmed that coating with GOQDs did not inhibit the generation of ^1^O_2_. We also confirmed the production of ^1^O_2_ by GOQDs using free HMONs as a control (Additional file 1: Fig. S7).

To study the drug loading and release profiles of HMONs@GOQDs, we used DOX as a model drug, and extracted it from doxorubicin hydrochloride (DOX·HCl) according to the procedure reported previously [63]. DOX-HMONs@GOQDs was obtained by adding DOX solution (5 mg/mL in dimethyl sulfoxide) during the coating procedure. As shown in Additional file 1: Fig. S9, the FTIR spectra of HMONs@GOQD, free DOX and DOX-HMONs@GOQDs were studied. The Si–OH groups of HMONs and -OH groups of GOQDs involved in adsorption interactions with water molecules produce the broad band around 3500-3000 cm^−1^. It overlaps the absorption peak related to the N–H stretching vibrations attributed to DN-based precursor and the chemical linkage between HMONs and GOQDs, the new peaks at 1567 cm^−1^ was attributable to the C = C of the GOQDs. Compared with HMONs@GOQDs, the new absorption peak at 1741 cm^−1^ of DOX-HMONs@GOQDs confirmed the successful physical adsorption of DOX. To evaluate the drug-loading capacity, we determined the concentrations of DOX before and after the loading process using a fluorescence spectrophotometer; the capacity was 112 ± 6 mg of drug per gram of HMONs@GOQDs. This high drug-loading capacity and efficiency (approximately 82%) due to the hollow structure will be beneficial for chemotherapy.

The HMONs@GOQDs are designed to be degraded by light irradiation, enabling the efficient release of the loaded cargo. To confirm this profile, we dispersed the DOX-HMONs@GOQDs in PBS buffer, and determined the concentrations of DOX after various illumination periods. As shown in Fig. 2e, less than 5% of the DOX was released after 3 h without any stimulus because GOQDs blocked the mesopores of HMONs. In contrast, following light irradiation there was a large increase in release (> 80% in 3 h), which was probably due to the degradation of HMONs by light. We also evaluated the release capability of the DOX-loaded free HMONs, and found that their release efficiency was much lower than that of the DOX-HMONs@GOQDs because the free HMONs were nondegradable.

**Scheme 3:**
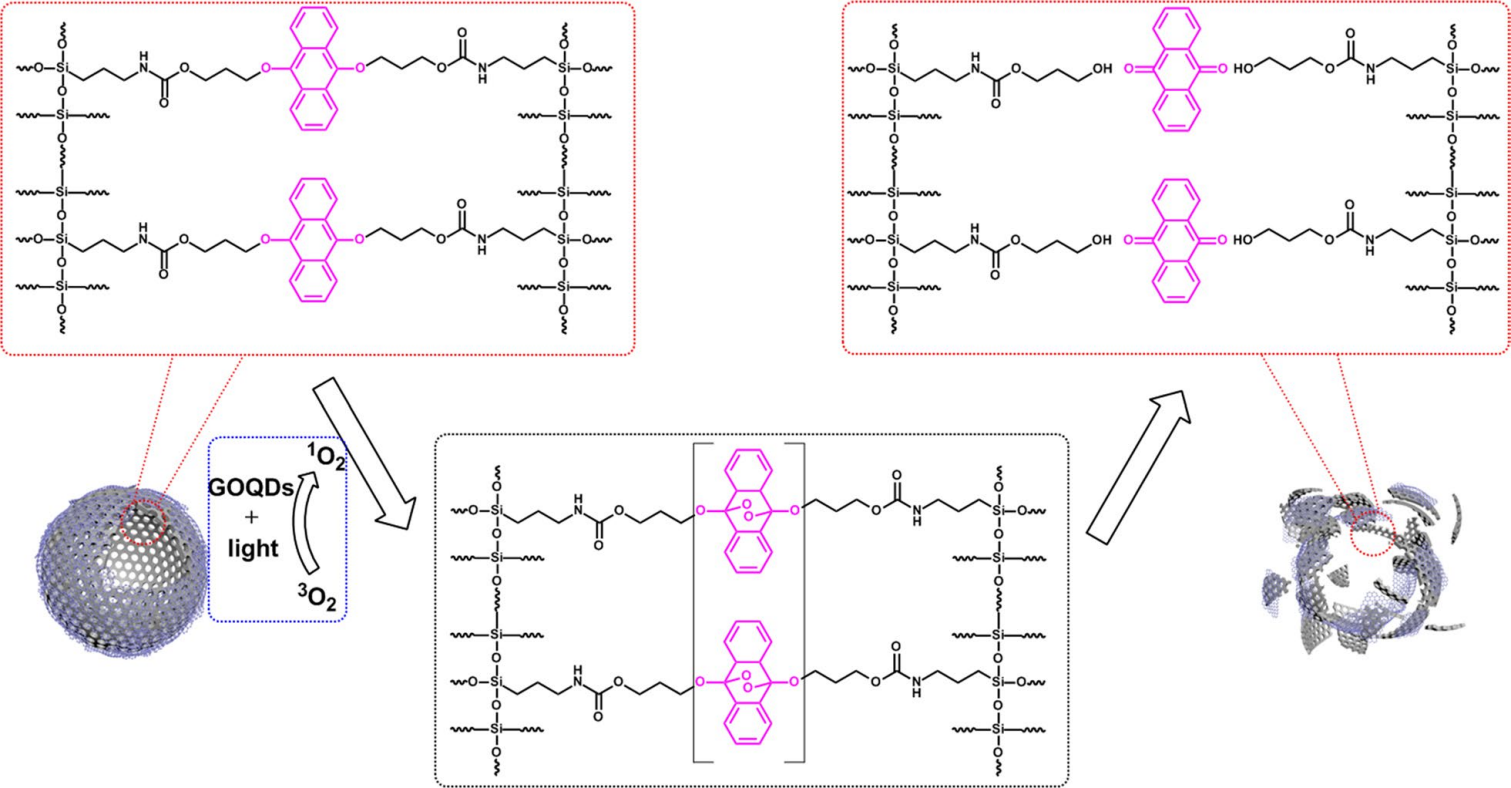
Schematic illustration of the degradation mechanism of HMONs@GOQDs

The degradation mechanism of HMONs@GOQDs was illustrated as Scheme 3. When irradiated with light, GOQDs produce ^1^O_2_, the DN groups in the in the shells of the HMONs would bind ^1^O_2_ efficiently and then react with ^1^O_2_ via the highly favorable [4 + 2] cycloaddition mechanism, forming stable endoperoxides which would spontaneously decompose in the follow-up proton-catalyzed reaction with the formation of products 9,10-anthraquinone (AQ), leading to the complete degradation of the HMONs [64].

**Fig. 3:**
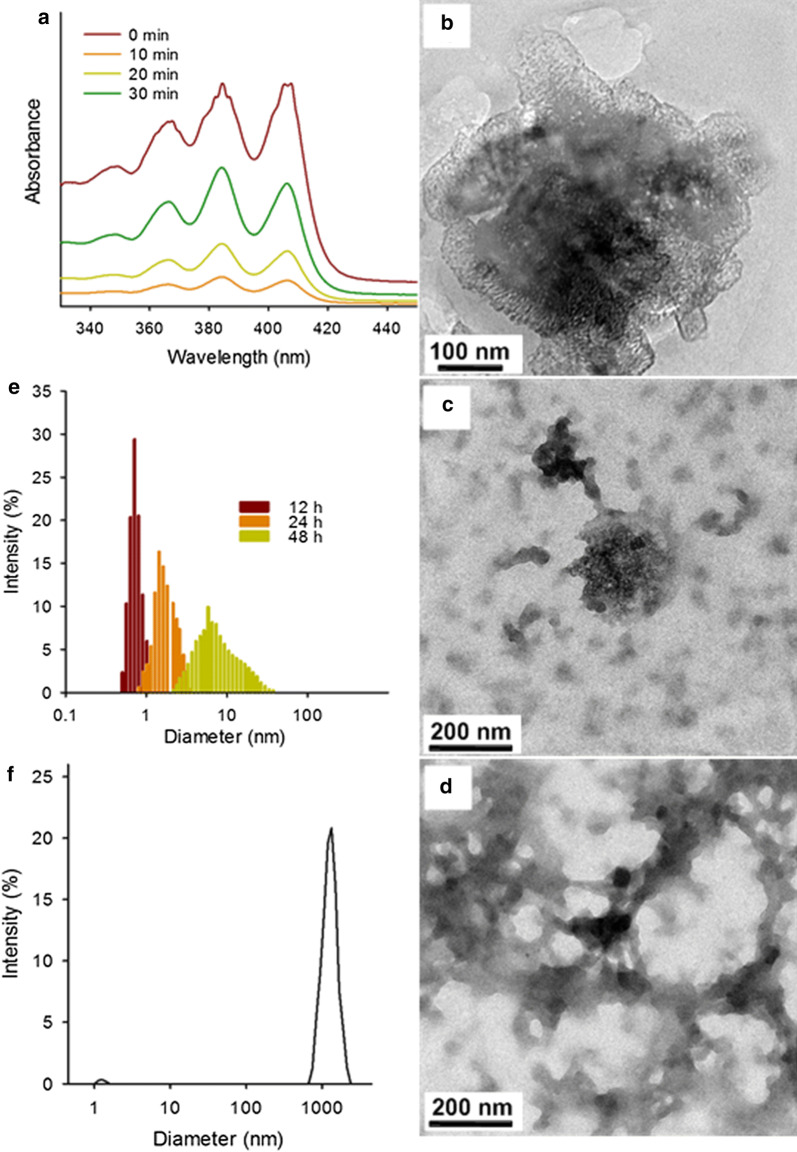
Degradation of HMONs@GOQDs. **a** UV–vis adsorption of the 9,10-dialkoxy-anthracene (DN) precursor following light irradiation for various periods; **b**–**e** (TEM images and size distribution of HMONs@GOQDs after various irradiation periods showing the structural evaluation of HMONs@GOQDs (12, 24, and 48 h); and **f** size distribution of the supernatant after centrifugation

To confirm this change, we mixed an ethanol solution of the DN precursor with the GOQDs, and irradiated it with light (365 nm, 0.8 W/m^2^) for various periods. The resulting UV–vis absorption spectra are shown in Fig. 3a. During light illumination for 0 to 30 min, the UV–vis absorption of the DN precursor decreased significantly with the generation of ^1^O_2_, confirming that the DN precursor was altered in the presence of ^1^O_2_.

The degradation of the developed nanosystem is of great significance in terms of its suitability as a drug carrier in a clinical context. We used TEM to monitor the structural evolution of HMONs@GOQDs during the degradation process stimulated by light irradiation (30 min per 12 h) over various periods (Fig. 3b-d). There was significant degradation and the nanosized particles were completely degraded after 48 h. We also recorded the particle-size distribution after 48 h (Fig. 3f). Following centrifugation, we collected a drop of the supernatant of the HMONs@GOQDs suspension, and further confirmed the presence of small fragments after exposure to light by means of DLS analysis (Fig. 3e). These results indicate that the HMONs@GOQDs are degraded by light irradiation, which should enable the efficient release of the loaded cargo. We also irradiated free HMONs with light for 48 h, and the resulting TEM image is shown in Additional file 1: Fig. S10. The small change in the structure of the HMONs further confirmed that the degradation of the HMONs@GOQDs was caused by photo-induced ^1^O_2_ generation. This observation also indicates that the nanosized particles will be safely excreted by the body once they have completed drug delivery.

**Fig. 4:**
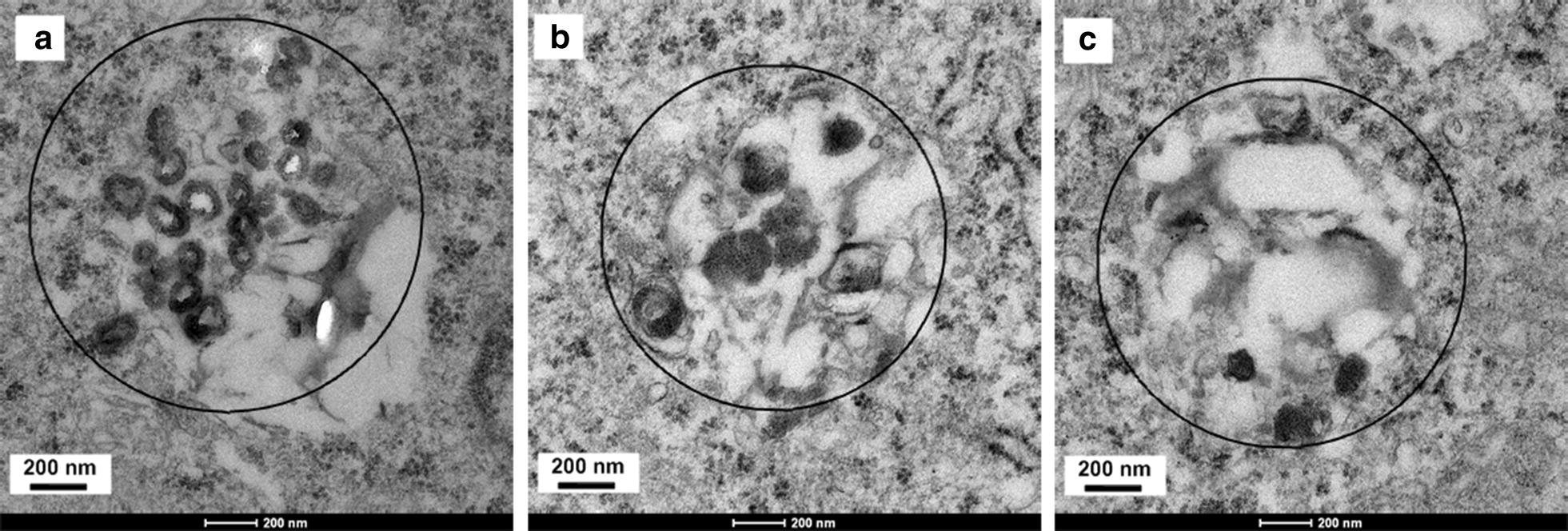
Bio-transmission electron microscopy (bio-TEM) images of the intracellular degradation and structural evolution of HMONs@GOQDs in 4T1 cancer cells following light irradiation for various periods (12, 24, and 48 h)

We further evaluated the degradation behavior and structural evolution of HMONs@GOQDs in cells. After co-incubating the HMONs@GOQDs with 4T1 cancer cells for 4 h, we irradiated the culture with light (365 nm, 0.8 W m^−2^, 30 min per 12 h, 2-min intervals for each 1-min irradiation). We harvested the cells after various periods, fixed them, and cut the resulting samples into ultrathin sections for bio-TEM investigation. As shown in Fig. 4a, the HMONs@GOQDs were efficiently endocytosed into the cancer cells; the hollow structures are clearly visible, and a small number of nanoparticles have degraded. Significant degradation is visible in Fig. 4b, and there are multiple amorphous morphologies. It should be noted that the vast majority of nanosized particles disappeared, demonstrating the complete degradation of the HMONs@GOQDs. The in vitro assays in both PBS solution and under intracellular conditions demonstrate the light-responsive degradation profile of the HMONs@GOQDs, which could potentially guarantee their controlled degradation in vivo.

**Fig. 5:**
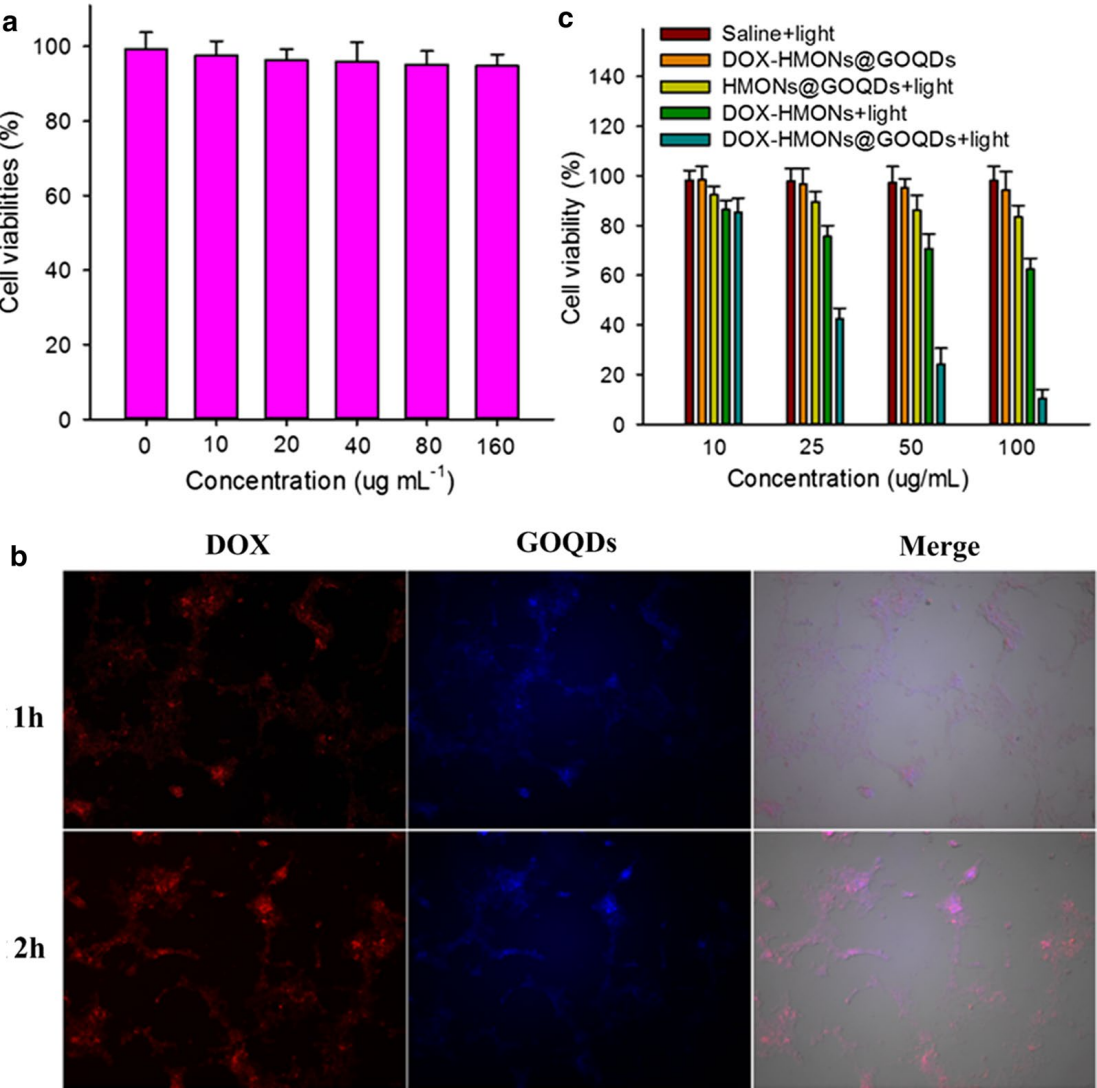
*In vitro* experiments on HMONs@GOQDs and DOX-HMONs@GOQDs. **a** In vitro cell viability of HMONs@GOQDs at various concentrations; **b** time-dependent cellular internalization of DOX-HMONs@GOQDs in 4T1 cells after incubation for 1 or 2 h; **c** phototoxicity of cells incubated with free HMONs@GOQDs, DOX-HMONs, or DOX-HMONs@GOQDs with or without laser irradiation

To confirm the biocompatibility of the HMONs@GOQDs, we first incubated the 4T1 cells with various concentrations of the HMONs@GOQD nanocarriers in PBS for 24 h, and determined the viabilities of the cells. As shown in Fig. 5a, the HMONs@GOQDs had very low cytotoxicity (cell viability > 90% after incubation for 24 h), even at a high dosage concentration (160 μg/mL). The results prove that both GOQDs and HMONs are biocompatible and nontoxic, which are necessary for further biomedical applications. We also incubated the 4T1 cells with various concentration of the original DN-based precursor in PBS for 24 h, and the resulting low cytotoxicity further confirmed the biocompatibility of the HMONs@GOQDs (Additional file 1: Fig. S11).

The uptake of HMONs@GOQDs by the cells demonstrated the nanocarriers’ ability to deliver drugs intracellularly. We investigated the distribution of the DOX-HMONs@GOQDs in the 4T1 cells using a fluorescence microscope to detect the fluorescence of DOX and GOQDs. It was obvious that the DOX-HMONs@GOQDs were efficiently taken up by cells (Fig. 5b).

The degradation of HMONs@GOQDs under light irradiation causes the leakage of the loaded drug at the molecular level, resulting in the destruction of the tumor cells. We incubated various concentrations of the DOX-HMONs@GOQDs with the 4T1 cells for 4 h, removed the excess nanoparticles, and further incubated the cells for 24 h in the dark. Viability was evaluated using a 3-(4,5-dimethylthiazol-2-yl)-2,5-diphenyltetrazolium bromide (MTT) assay with or without light irradiation. We used 4T1 cells treated with saline and DOX-HMONs under light irradiation as control groups. Compared to the DOX-HMONs@GOQDs in the absence of light irradiation and the control groups, the irradiated DOX-HMONs@GOQDs has significantly enhanced cytotoxicity with regard to the cancer cells. This further confirmed the photo-controlled release and significant accumulation of DOX inside the cells owing to the degradation of the HMONs@GOQDs. The distribution of DOX was further demonstrated using co-localization fluorescence measurements after 4T1 cells were stained with DAPI. As shown in Additional file 1: Fig. S12, the fluorescence of DOX was mainly distributed in cytoplasm without light irradiation and nucleus with light irradiation, confirming the DOX leakage form the nanocarriers.

We also incubated the 4T1 cells with free HMONs@GOQDs, and the light-induced production of ^1^O_2_ also resulted in cytotoxicity with regard to the cancer cells. However, this cytotoxicity was much lower than that of light-stimulated DOX-HMONs@GOQDs owing to the low content of GOQDs. Therefore, the cytotoxicity of the DOX-HMONs@GOQDs was mainly caused by the significant accumulation of DOX resulting from the degradation of the HMONs.

It is worth noting that nano-sized HMONs@GOQDs can accumulate passively in tumor cells through the enhanced permeability and retention (EPR) effect, and ^1^O_2_ is only generated during light irradiation. Therefore, the generation of ^1^O_2_ does not have a toxic effect on normal tissues [52, 61].

**Fig. 6:**
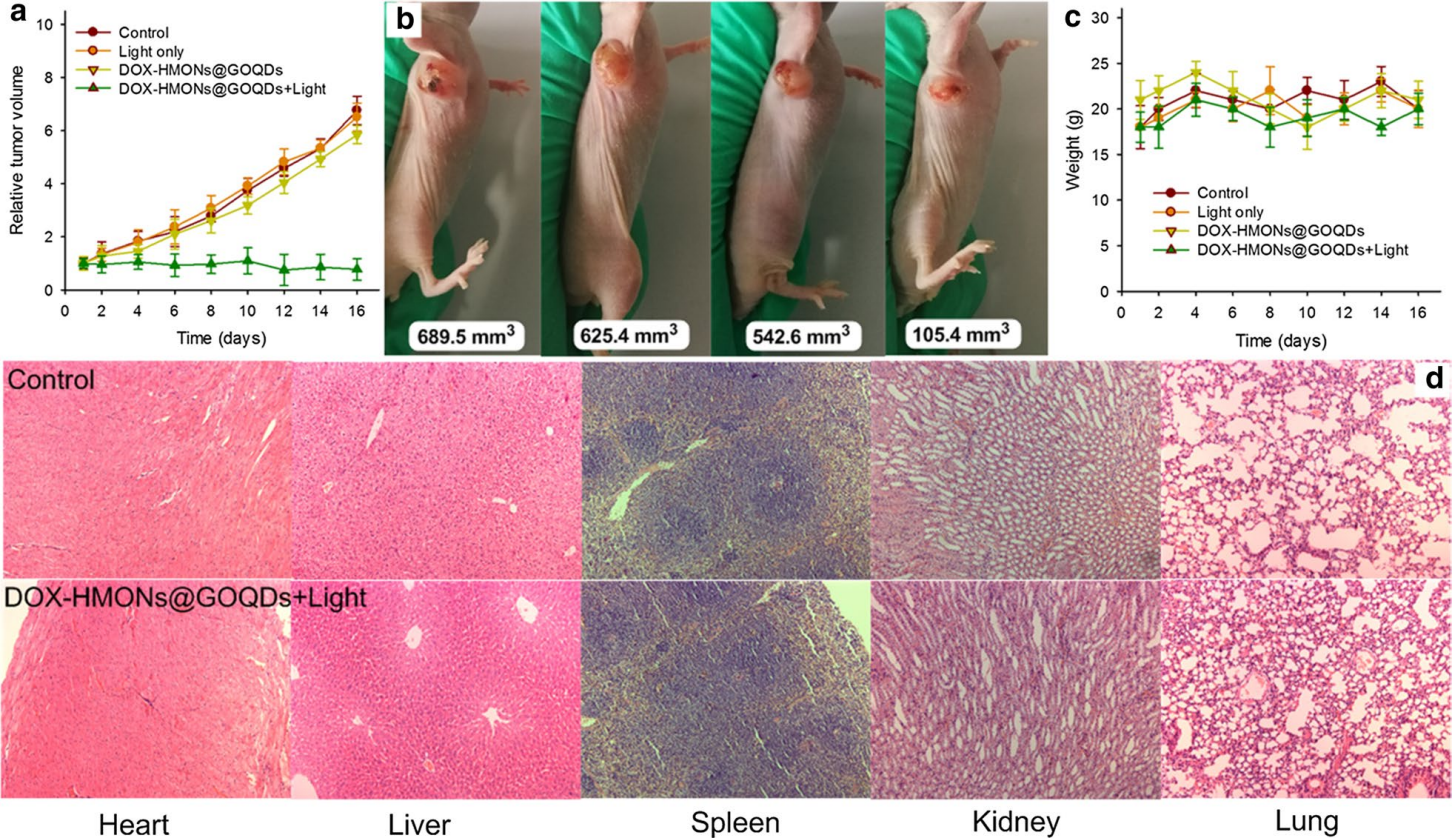
In vivo antitumor efficacy of DOX-HMONs@GOQDs. **a** Growth curves of 4T1 tumors in each group; **b** photographs of tumors at the end of each treatment; **c** changes in the body weights of the mice over the treatment period; **d** haemotoxylin and eosin (H&E) staining in the normal tissue group (the control) and the DOX-HMONs@GOQDs group

To study the antitumor activity of the DOX-HMONs@GOQDs during light irradiation in vivo, we randomly divided male athymic nude mice bearing 4T1 cells into four groups: a control group, a light only group, a free DOX-HMONs@GOQDs group, and a DOX-HMONs@GOQDs + light group. The DOX dosage was equivalent to the clinical dosage given to patients (5 mg kg^−1^), and the concentration of DOX was ca. 1.6 mg mL^−1^. Our estimate of therapeutic efficiency was based on the change in tumor volume. As illustrated in Fig. 6a, the light-irradiated DOX-HMONs@GOQDs significantly inhibited tumor growth, whereas tumor growth was not affected in the light irradiation only and free DOX-HMONs@GOQDs groups. Photographs of the tumors at the end of each treatment also revealed that the tumors were smaller in the DOX-HMONs@GOQDs + light group than in the other three groups (Fig. 6b). These results suggested that the tumor cells are able to take up the DOX-HMONs@GOQDs via an endocytosis pathway, and the loaded DOX could be released from the HMONs@GOQDs under light irradiation, and they further confirmed the photo-responsive degradation profile of HMONs@GOQDs. We also used male athymic nude mice bearing 4T1 cells—which had been treated with a mixture of the DN-based precursor and GOQDs—as a control group. The GOQDs, the original DN-based precursor, and the degradation products did not inhibit tumor growth. And then another group was received with free DOX at an equivalent DOX dose of 5 mg/kg, free DOX also could inhibit tumor growth, however, the administration of DOX-HMONs@GOQDs + light exhibited much higher tumor-inhibition effect compared to free DOX, further confirming that the significant effects in the DOX-HMONs@GOQDs + light group were caused by accelerated drug release (Additional file 1: Fig. S13). In addition, we fixed tumor cells from the tumor tissues, and cut them into ultrathin sections for bio-TEM investigation at 12 h after treatment injection. The HMONs@GOQDs were clearly visible in the cancer cells (Additional file 1: Fig. S14), indicating that the administrated nanoparticles did find their way into those cells in vivo. As shown in Fig. 6c, each mouse retained its weight well over the entire treatment period, indicating that both the DOX-HMONs@GOQDs and the other treatments would be safe for topical in vivo applications. To further confirm the safety of the treatments, we collected the major organs and subjected them to haemotoxylin and eosin (H&E) staining. As shown in Fig. 6d, treatment with DOX-HMONs@GOQDs and light had little impact on the liver or on the other major organs.

## Conclusions

In conclusion, we developed novel photo-responsive biodegradable mesoporous organosilica nanoplatforms (HMONs@GOQDs) for anticancer drug delivery. The HMONs in the HMONs@GOQDs are based on ^1^O_2_-responsive DN-bridged organoalkoxysilanes, and degrade when irradiated with light owing to the GOQDs on their surfaces. The well-defined hollow mesoporous structures of the HMONs@GOQDs provide high drug-loading capacity, and photo-responsive degradation endows the nanoplatforms with photo-controlled drug release profiles. We carried out in vitro and in vivo experiments, which demonstrated the excellent therapeutic efficacy of the HMONs@GOQDs. Moreover, the degradability and clearance of the nanoplatforms avoid long-term toxicity, thereby qualifying them for cancer therapy clinical trials. However, short wavelength light (365 nm) does not readily penetrate the thick tissues of human patients. Nevertheless, the as-prepared HMONs@GOQDs have extensive application potential for cutaneous tumor therapy. Moreover, nitrogen-doped GOQDs or upconversion materials might be used to increase the range of light adsorption and expand the applicability of the HMONs@GOQDs in the future.

## Supplementary information

**Additional file 1.** Additional figures.

## Data Availability

All data generated or analyzed during this study are included in this published article.
