# Supplementary material for: Photo-responsive degradable hollow mesoporous organosilica nanoplatforms for drug delivery
Source: J Nanobiotechnology. 2020 Jun 15;18:91. doi: 10.1186/s12951-020-00642-1 (PMC7296706; doi:10.1186/s12951-020-00642-1)
Supplement: Supplementary file 1 — Additional file 1. Additional figures. [file 12951_2020_642_MOESM1_ESM.doc]

Photo-responsive degradable hollow mesoporous organosilica nanoplatforms for drug delivery

Jie Fan, Zhipeng Zhang, Yaru Wang, Shiting Lin and Shun Yang*

School of Chemistry and Materials Science, Jiangsu Normal University, Xuzhou, Jiangsu, 221116, China


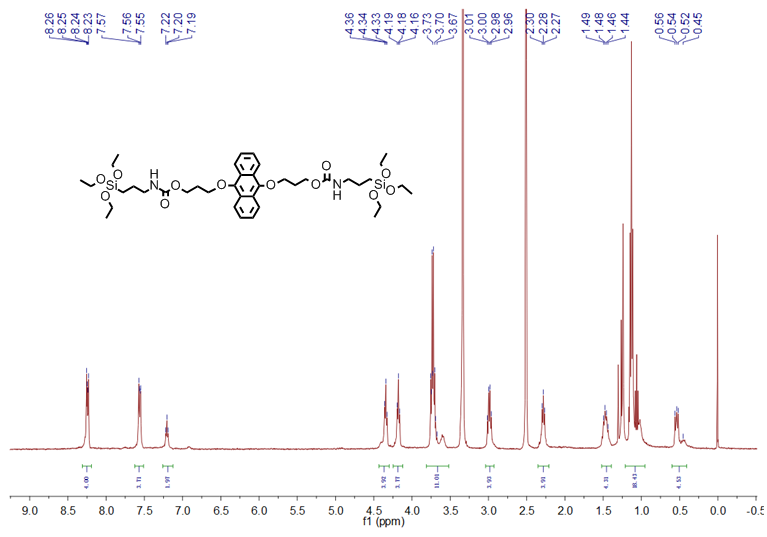


**Fig. S1** ^1^H NMR spectrum of 9,10-dialkoxy-anthracene (DN) based precursor .


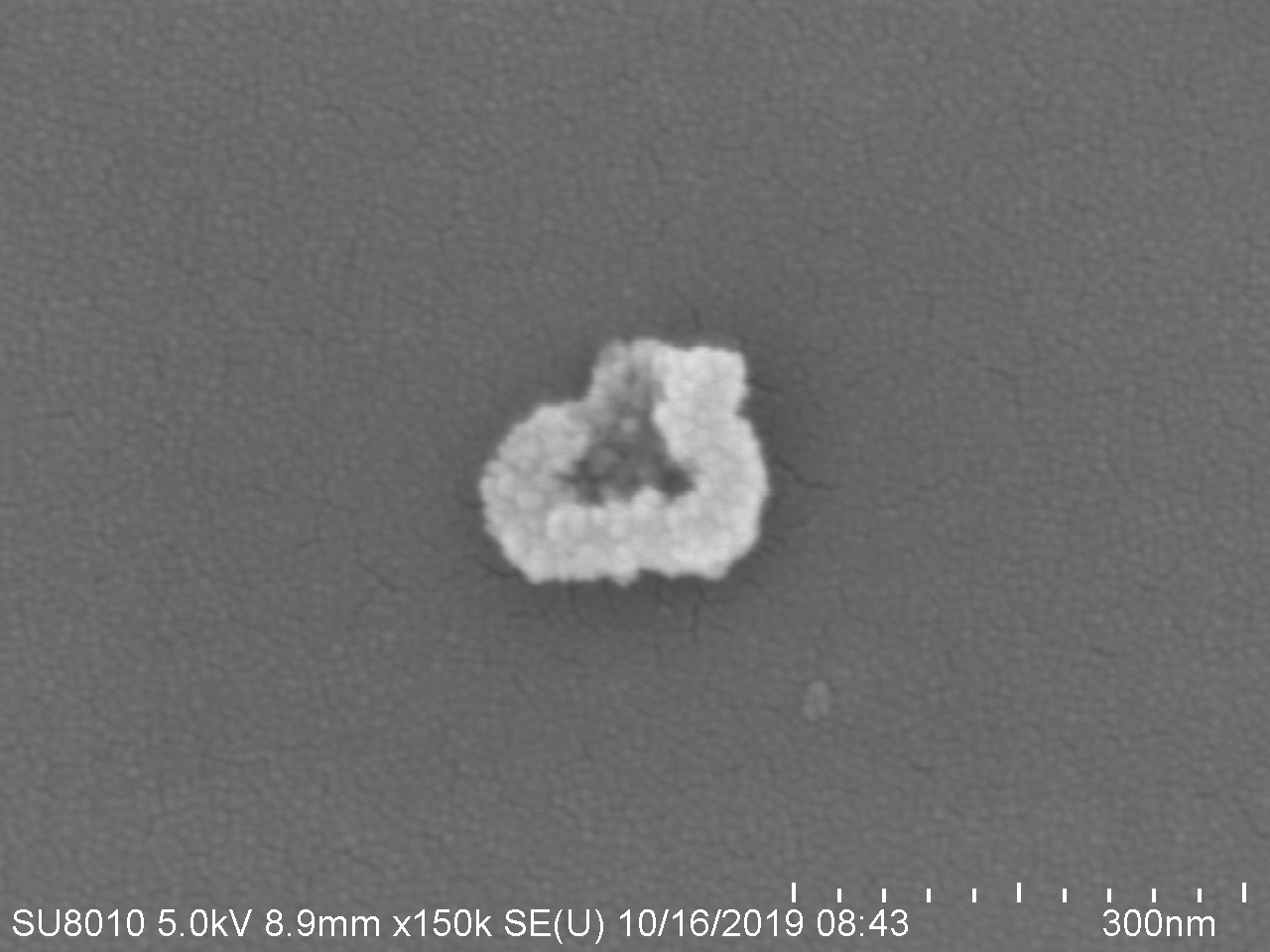


**Fig. S2** SEM image of a broken HMONs.


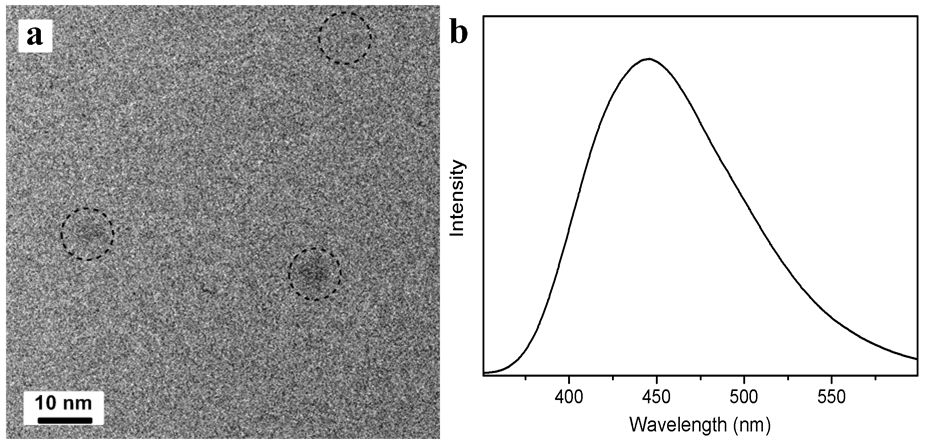


**Fig. S3** TEM image and emission spectrum of GOQDs.


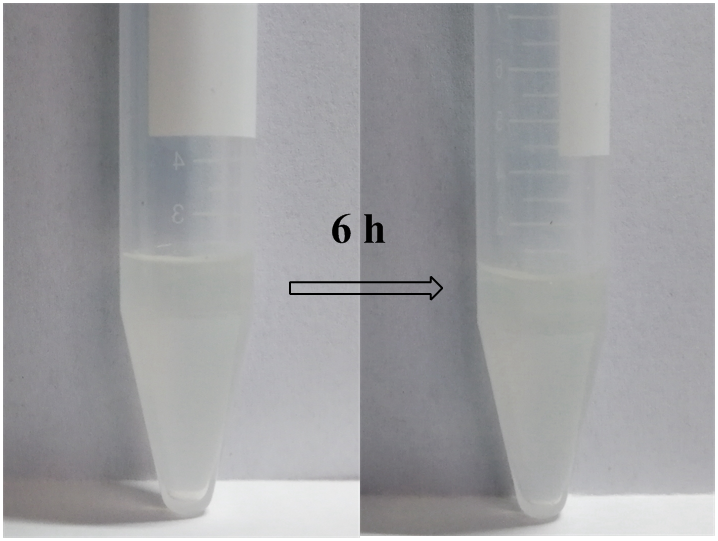


**Fig. S4** Photographs showing the dispersion of HMONs@GOQDs in PBS at 25 ^o^C

**
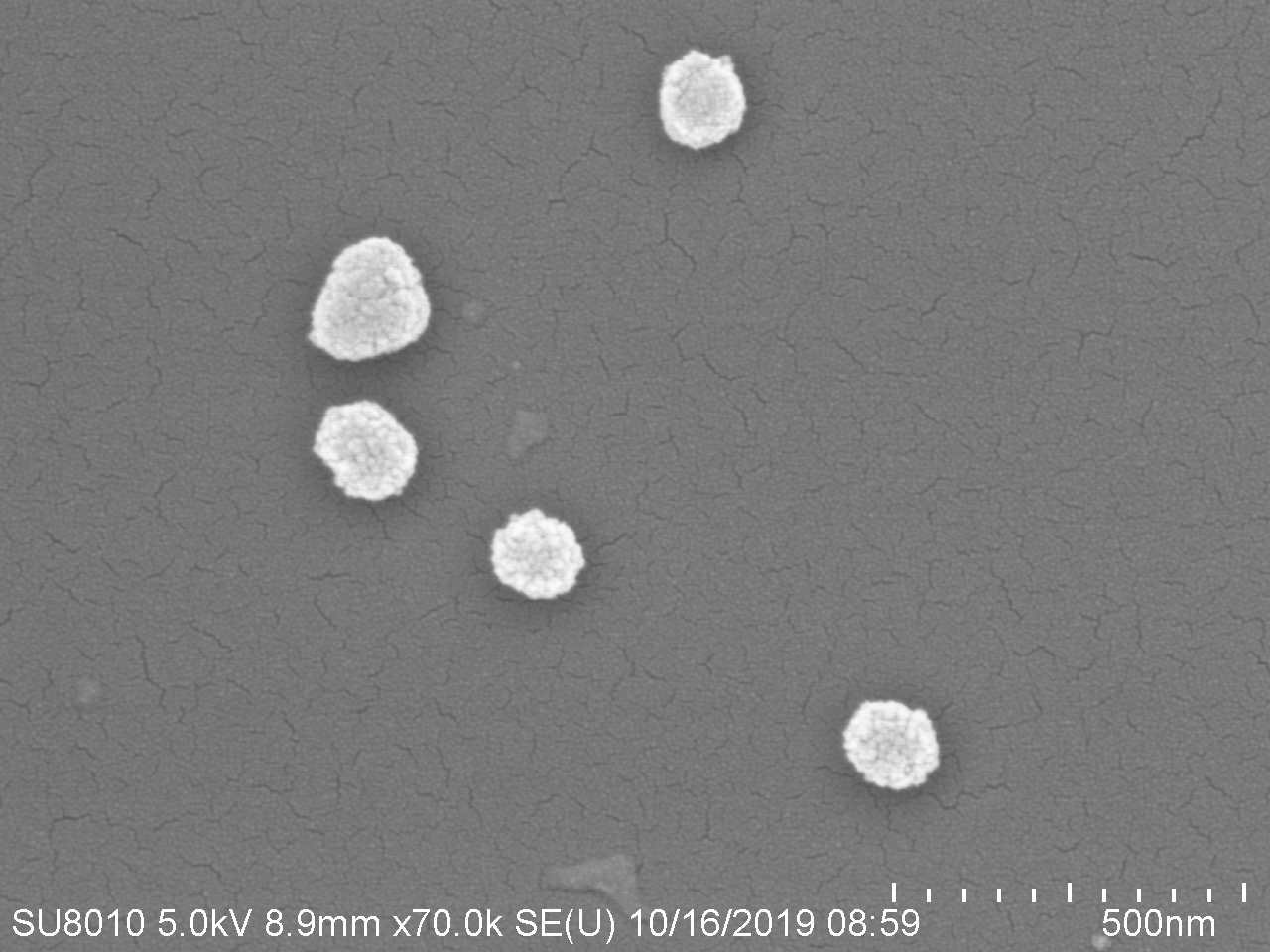
**

**Fig. S5** SEM image of a diluted HMONs@GOQDs sample.


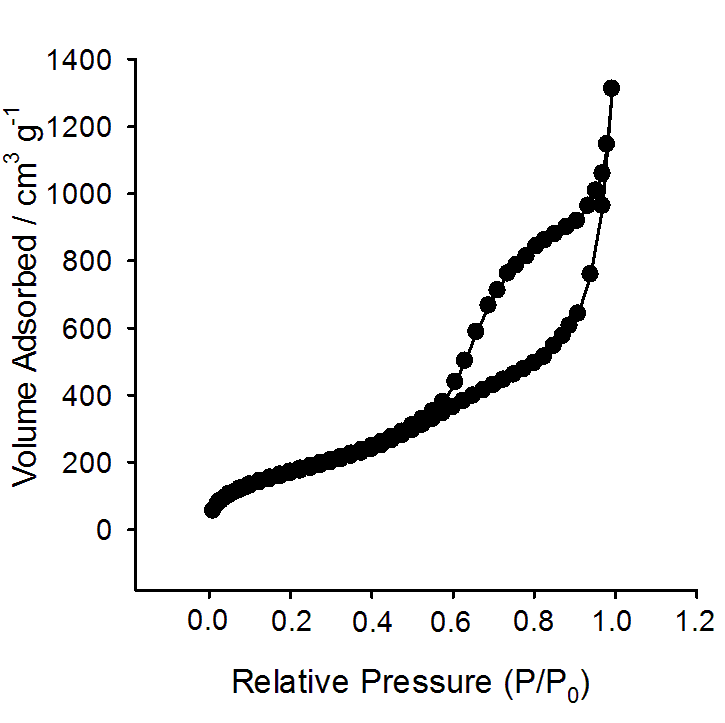


**Fig. S6** N_2_ adsorption and desorption pattern of the HMONs@GOQDs


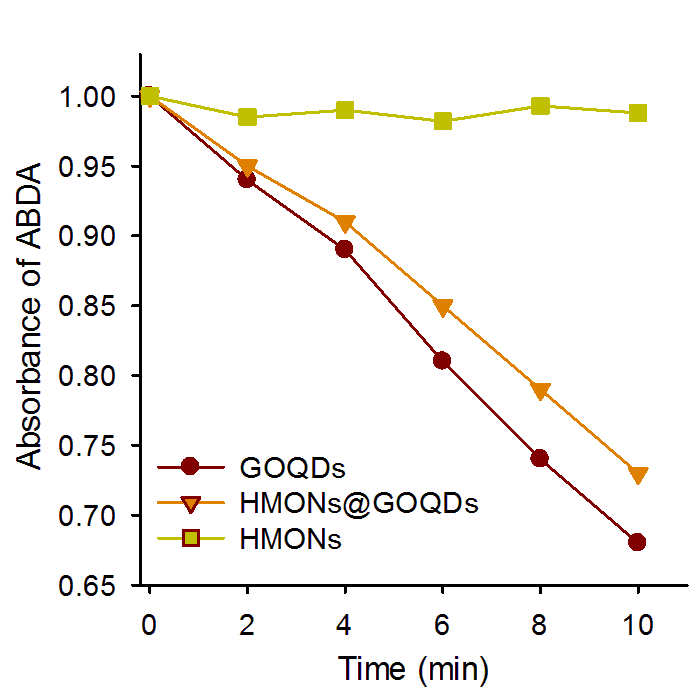


**Fig. S7** The generation of singlet oxygen by GOQDs, HMONs@GOQDs and free HMONs.


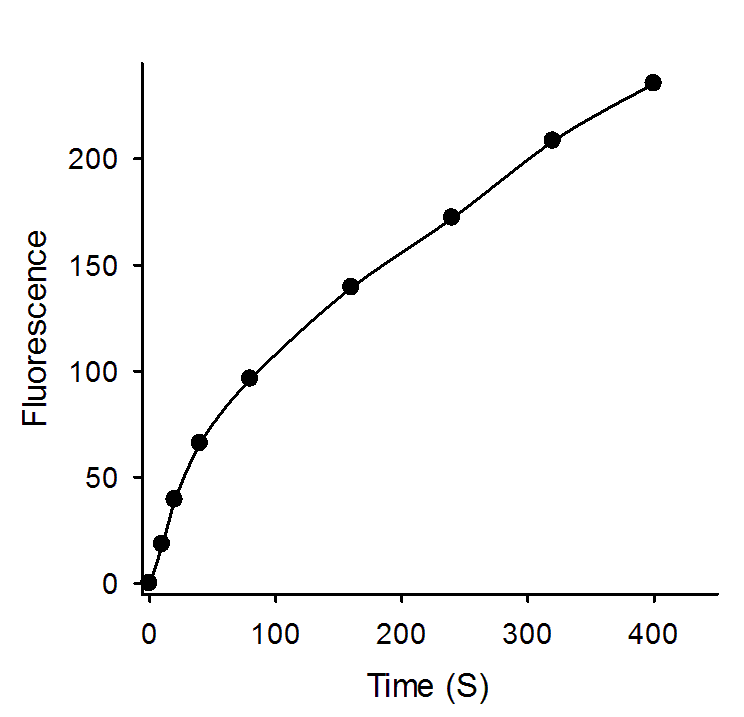


**Fig. S8** The ^1^O_2_ generation of GOQDs detected by SOSG assay.


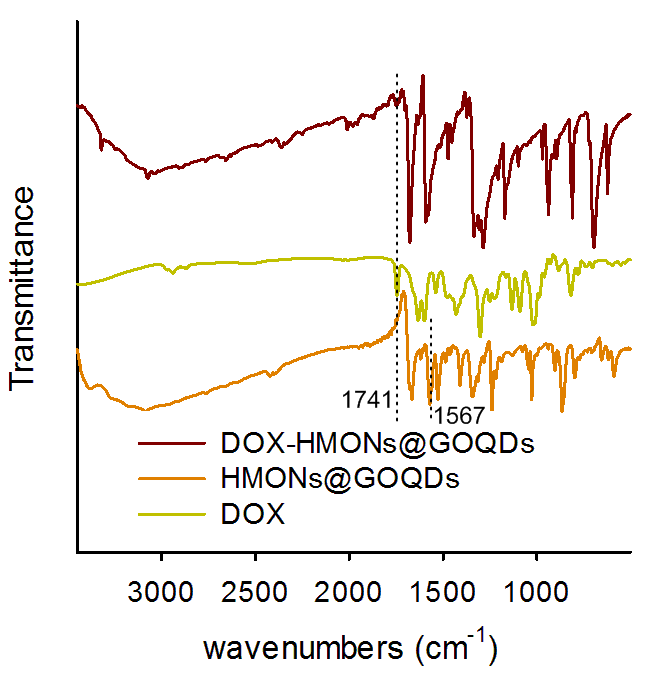


**Fig. S9** Fourier-transform infrared (FTIR) spectra of free DOX, HMONs@GOQDs and DOX- HMONs@GOQDs.

**
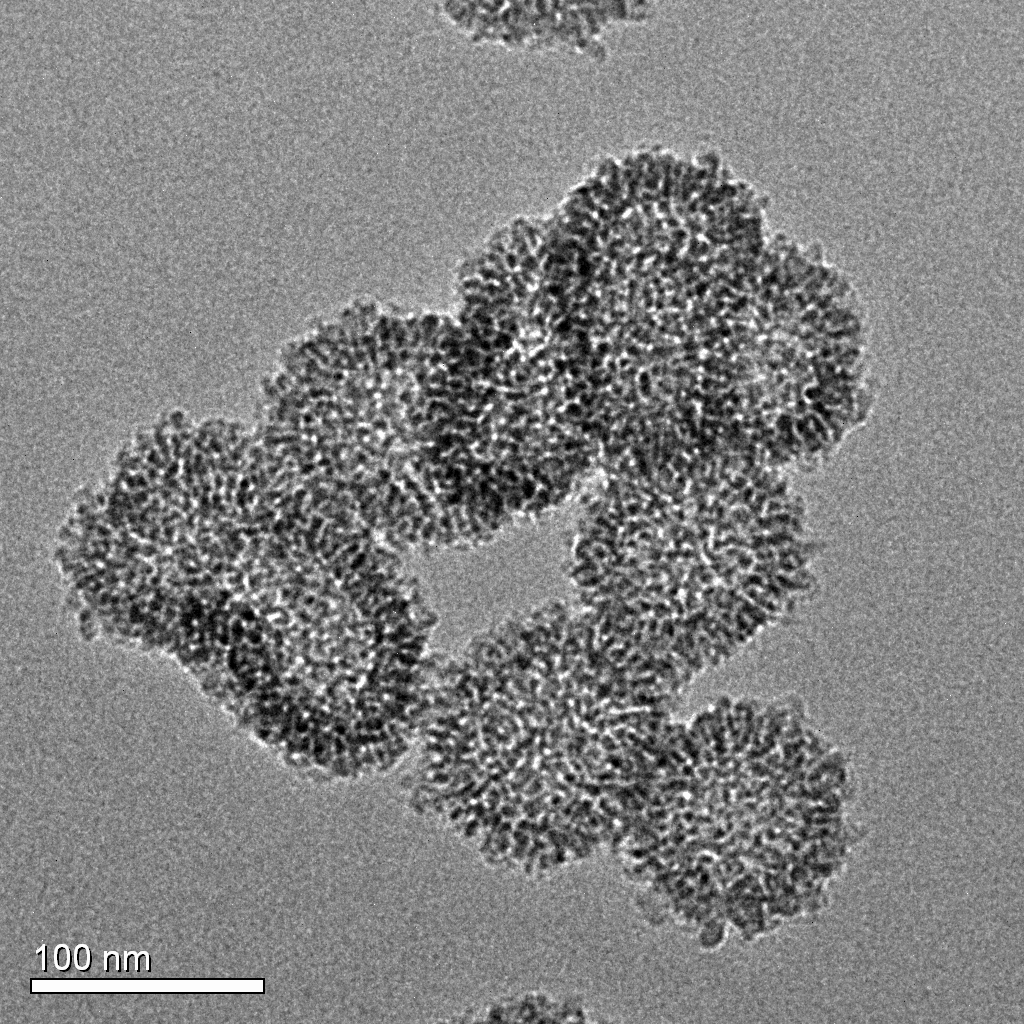
**

**Fig. S10** TEM image of free HMONs with light irradiation for 48 h.


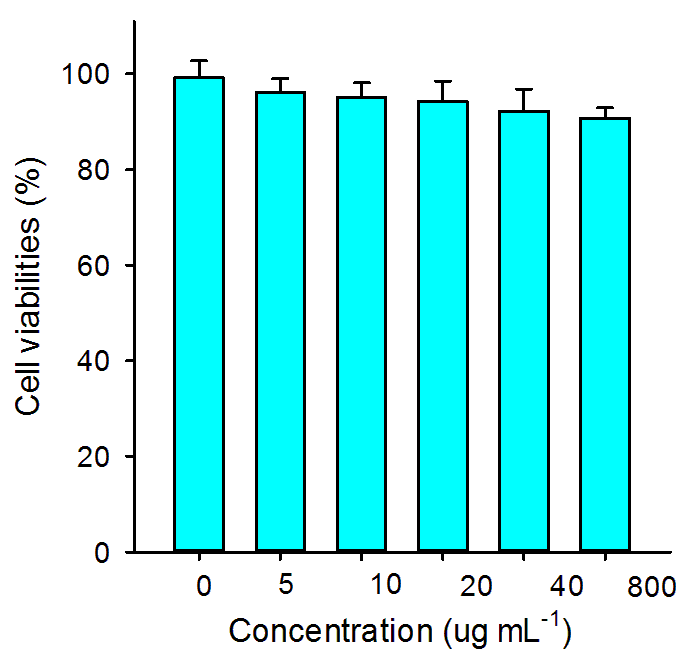


**Fig. S11** In vitro cell viability of 9,10-dialkoxy-anthracene (DN) based precursor at different concentrations.

**
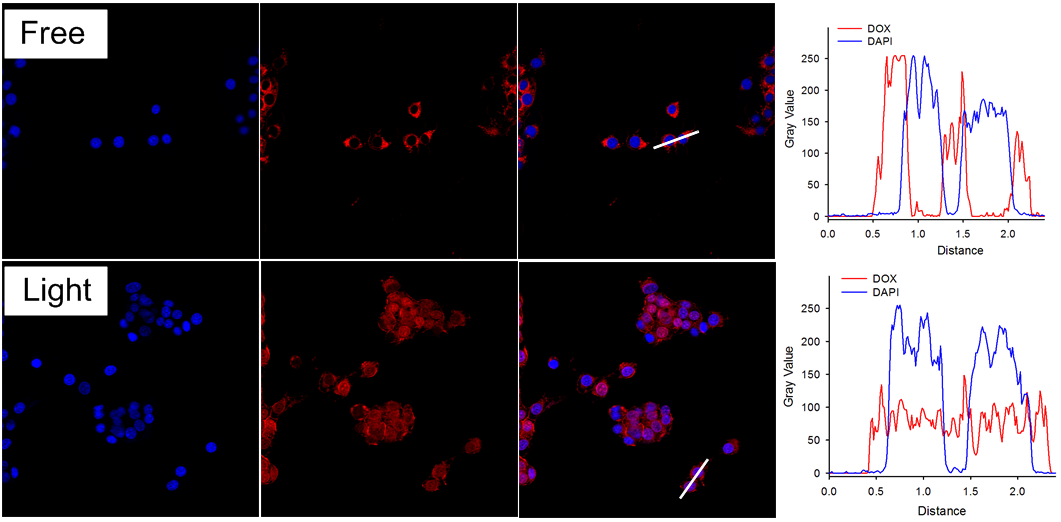
**

**Fig. S12**. Co-localization fluorescence measurements of 4T1 cells incubated with DOX-loaded PBHMONs with and without light irradiation.


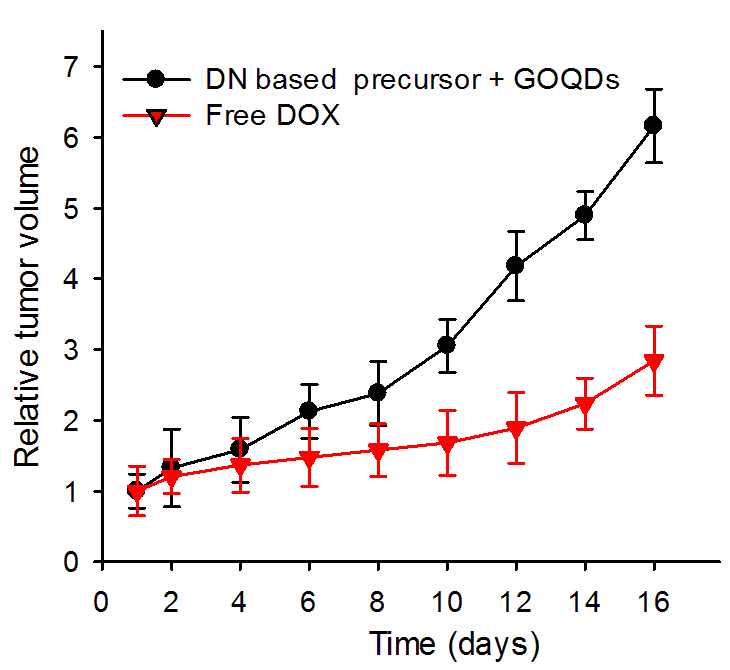


**Fig. S13** Growth curves of 4T1 tumors in 9,10-dialkoxy-anthracene (DN) based precursor and GOQDs treated group and free DOX treated group.


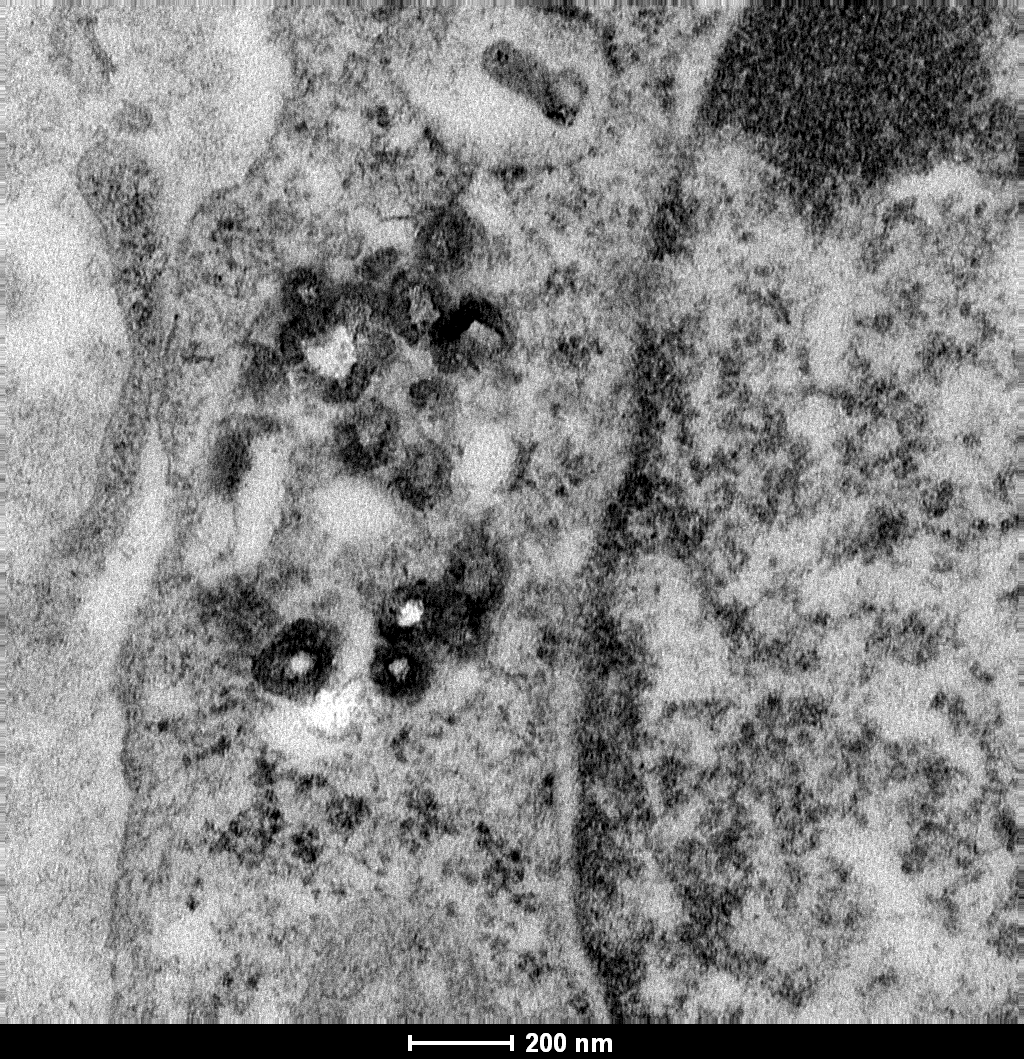


**Fig. S14** TEM image of tumor cells from tumor tissues.
